# Harnessing the healing power of nature: a review of natural interventions in substance abuse treatment and prevention

**DOI:** 10.1265/ehpm.24-00145

**Published:** 2024-11-12

**Authors:** Francisco Díaz-Martínez, Miguel F Sánchez-Sauco, Laura T Cabrera-Rivera, Claudia A Ortín-Fernández, Esteban Orenes-Piñero, Juan A Ortega-García

**Affiliations:** 1 Paediatric Environmental Health Specialty Unit, Department of Pediatrics, Clinical University Hospital Virgen of Arrixaca, University of Murcia, 30120 Murcia, Spain; 2 Environment and Human Health Lab (EH2), Instituto Murciano de Investigación Sanitaria (IMIB), University of Murcia, 30120 Murcia, Spain; 3 Global Alliance for Rewilding Child and Adolescent Health (GreenRooting.org), Spanish Association of Pediatrics, Madrid, Spain; 4 Department of Environmental Health, University of Puerto Rico, Medical Sciences Campus, San Juan, PR 00921, US; 5 Department of Pediatrics, Hospital Adriana Cousiño de Quintero, Valparaíso, Chile; 6 Children’s Environmental Health Committee, Chilean Society of Pediatrics, Santiago, Chile; 7 Department of Biochemistry and Molecular Biology, University of Murcia, IMIB Arrixaca, Murcia, Spain

**Keywords:** Nature contact, Green space, Blue space, Disorder management, Substance abuse, Drug abuse

## Abstract

**Background:**

Substance abuse is a global problem that cuts across all sectors of society and requires innovative solutions that go beyond conventional treatments. Contact with nature could be a complementary tool to address drug-related problems. This review aimed to assess the impact of natural environments on drug-related outcomes.

**Method:**

8205 articles were screened between 2013 and 2023 from 6 databases, of which 21 met the inclusion criteria.

**Results:**

Most studies (12) focused on treatment, followed by incidence/consumption (7), prevention (5) and mortality (1). The main drugs studied were drugs in general (12), followed by alcohol (6), tobacco (6), and other drugs, including cannabis and opioids (4). The results of 85% of the studies showed positive outcomes, supporting the effectiveness of nature-based interventions for drug dependence. While some studies produced neutral or negative results.

**Conclusion:**

The use of nature-based interventions for the prevention and treatment of drug addiction shows considerable potential. However, more research is needed to understand the underlying mechanisms and to improve evidence-based interventions. Integrating health and environmental policies is essential to promote a holistic approach to drug strategies at the national and international levels.

**Supplementary information:**

The online version contains supplementary material available at https://doi.org/10.1265/ehpm.24-00145.

## Introduction

Globally, the consumption of illegal drugs, alcohol, and tobacco are the main contributors to the global burden of morbidity worldwide. Special attention is required for the mental health problems associated with this issue, which transcends geographical, social, and economic borders. According to the United Nations Office on Drugs and Crime (UNODC), in 2023, it is estimated that 296 million people worldwide consumed drugs, a 23% increase compared to the previous decade [1]. In Europe, recent estimates suggest that at least a quarter of the European Union (EU) population has used illegal drugs, and more than 4% of the population is affected by alcohol or drug dependence [2]. Moreover, the number of people suffering from drug use disorders has increased to 39.5 million, representing a 45% increase in 10 years [1]. According to the World Health Organization (WHO), drug consumption ranks among the primary causes for the reduction in healthy life years globally, attributable to both premature mortality and disability [3]. Despite the difficulty in assessing the social and economic costs associated with the use of legal and illegal drugs, in Europe, the cost is estimated to be between 0.1% and 0.4% of the Gross Domestic Product (GDP) for illicit drugs, 0.3% and 1.2% of GDP for tobacco, and alcohol, the figures were even higher despite enormous variability, with most studies exceeding 1% of GDP and some between 2% and 3.5% [4]. In order to contextualise this, it is notable that the total expenditure on healthcare in Europe typically represents approximately 11% of GDP. This indicates that the costs associated with alcohol, tobacco and other drugs consumption are considerable in comparison to other major economic expenditures [5]. In terms of mortality, drug use is responsible for approximately 16.8 million deaths each year, with over 70% of these deaths linked to opioid consumption [6]. However, it is essential to highlight that these data could be underestimated due to factors such as social stigma, limitations in data collection, and possible underreporting of use in certain population groups [7].

The phenomenon of drug use has been the subject of attention and concern globally, given its impact on the health of individuals [8]. The devastating consequences of substance use problems, which affect both individuals and society as a whole [9], have prompted the constant search for effective preventive and therapeutic approaches. Ongoing research and the continuous search for factors that may influence the spheres related to drug use play a crucial role in mitigating this public health issue [10]. Despite significant efforts to address this problem, traditional approaches to consumption, prevention, treatment, and rehabilitation have shown barriers in effectiveness and validity in the face of the challenges [11]. The need for innovative and complementary approaches to address addiction has become an urgent priority, prompting researchers to pursue novel solutions.

As part of this search, contact with nature, in a broad sense, or nature-based therapies have emerged as a promising field of research and work that offers a novel approach to addressing addiction challenges [12]. With its ecosystem values and intrinsic therapeutic benefits, nature has become a valuable resource in promoting mental health. It has been proven helpful in improving depressive mood, reducing anxiety, enhancing positive affect, and diminishing adverse effects [13]. The idea that exposure to natural environments can positively influence the prevention, treatment, and rehabilitation of individuals with substance use issues has caught the attention of health professionals, researchers, and policymakers [14]. Nevertheless, despite the growing scientific evidence supporting the mental health benefits of nature [15], there remains a significant gap in our knowledge regarding its potential influence on the spheres related to substance use.

This review aims to synthesize the available scientific evidence on the impact of wilderness exposure on individuals affected by substance use. We aim to provide a comprehensive view of how nature can contribute to consumption reduction, prevention, treatment, and rehabilitation in the context of substance abuse and to promote tools and knowledge applicable to public health and environmental policies.

## Materials and methods

### Search strategy and information sources

This review explores the relationship between exposure to natural spaces and various aspects of drug use, including prevention, treatment, and incidence. Our approach was designed to provide a comprehensive overview of this field’s current state of knowledge.

**Fig. 1:**
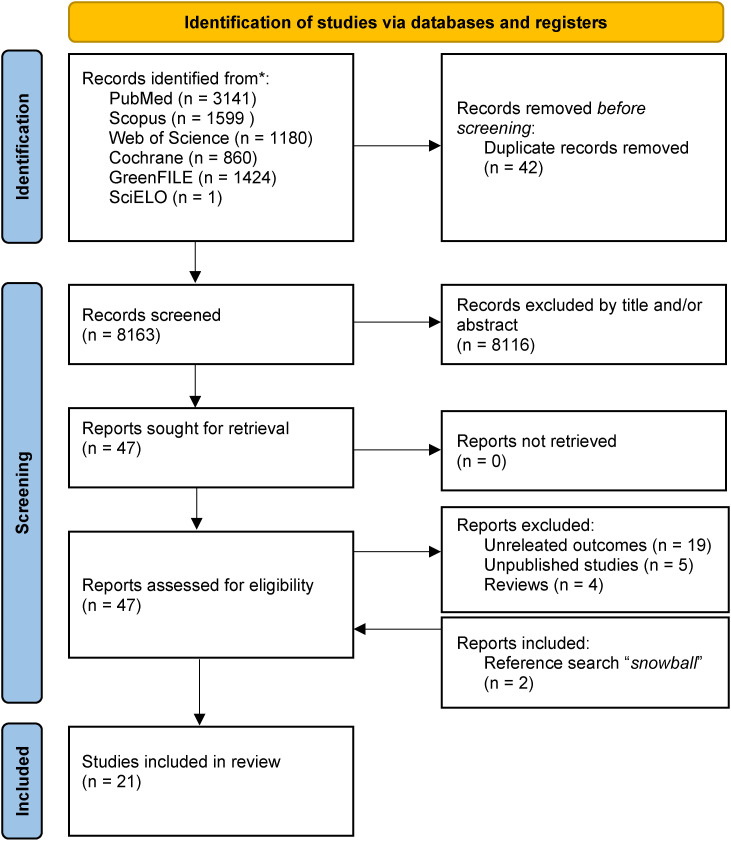
PRISMA-P Flowchart of the stepwise assessment of articles obtained from the search strategy.

In October 2023, we conducted an extensive literature search using multiple databases: PubMed, Scopus, Cochrane, GreenFILE, Web of Science, and SciELO. Keywords were selected based on recent literature in the field, focusing on terms related to natural spaces and drug use. Our search strategy was iterative, allowing for the inclusion of additional relevant sources as they were identified during the review process. The search strategy and analysis methods were recorded in the PROSPERO database under a registered protocol (ref: CRD42023465448). Three reviewers performed the searches in the electronic databases mentioned beforehand. The studies identified in the different searches were combined, duplicates were removed, and articles were reviewed based on their title and abstract content to determine their relevance for the review. New articles were found through the citations of the included publications using the ‘snowball effect’ strategy (Fig. 1). Different searches were conducted in the databases used, connecting with the Boolean operators “AND” and “OR” using Mesh terms (“Parks, recreational”, “Relaxation Therapy”, “Horticultural Therapy”, “Substance-Related Disorders”, “Substance Abuse Detection”, “Smoking Prevention”, “Alcoholism”, “Cocaine-related disorders”, “Substance abuse Treatment Centers”, “Marijuana abuse”, “Psychotropic Drugs”, “Opioid-Related Disorders” and “Harm Reduction”) and free terms (“Drug use”, “Substance abuse”, “Addiction treatment”, “Addiction prevention”, “Addiction rehabilitation”, “psychoactive substance use”, “drugs”, “substance”, “alcohol”, “tobacco”, “smoke”, “marijuana”, “cocaine”, “opioids”, “people with drug problems”, “recreational drug users”, “drug dependents”, “patients in addiction treatment”, “problematic substance use”, “harm reduction”, “substance use disorder”, “garden therapy”, “natural environments”, “exposure to nature”, “forest”, “contact with nature”, “effects of nature on health”, “greenspace”, “Green space”, “greenness”, “outdoor therapy”, “ecotherapy”, “nature-based therapy”, “nature-based interventions”, “outdoor activities”, “exposure to vegetation”, “connection with the natural environment”, “wilderness therapy”, “rehabilitation in natural environments”, “Green care”, “Green exercise”, “Green prescription”, “forest bath”, “psychological ecosystem services”) in different combinations. A filter was also used to obtain results from January 2013 to October 2023. Articles were searched in English and Spanish, although only English articles were found.

### Inclusion and exclusion criteria

The studies were evaluated by three reviewers and included if: (1) they assessed the relationship between exposure to natural spaces* and some aspect of drug use**; (2) they used some qualitative or quantitative measure to evaluate aspects related to the incidence, mortality, prevention, or treatment of drug use; (3) the articles were written in English or Spanish. Excluded articles were those that had results not related to the objectives of the review, the data related to drug use were not directly related to the study population, were systematic or narrative reviews or meta-analyses, did not specify the population of interest, and those studies with unpublished results in indexed scientific journals.

*Natural spaces are defined as all those outdoor spaces, whether natural or artificial, that prominently feature vegetation and/or water and are accessible to humans either proximately (being in, within, or near the vegetation and/or water) or virtually (being able to see, hear, or otherwise sense nature). **Drugs and/or psychoactive substances are defined as natural or synthetic compounds that act on the nervous system, causing alterations in functions that regulate thoughts, emotions, and behavior [16]. Studies referring to “medical drug” or “medicament” were not incorporated with the term “drugs”.

### Identification of studies and data analysis

All selected references were included in their complete manuscript form. The review team collaboratively resolved any ambiguities regarding article inclusion. The data extracted from each article included reference and publication year, geographical location, objective and hypothesis, study design, sample size, project/study framework, study population, type of exposure to natural spaces, source of exposure data, sphere or domain of drug use affected, type of drug affected, evaluation tool, statistical methodology, adjustment/confounding factors, main findings, benefit categorization (positive/negative), and limitations/strengths. To facilitate the interpretation of the results and simplify the tables, the manuscript only presents the bibliographic reference of the studies, geographic location, design, sample size, population, type of exposure to nature and data source, domain or sphere related to drug use and drug type, assessment tools and data source, the relationship between variables and main outcomes.

### Quality assessment

The quality assessment of the studies included in the review was based on the Quality Assessment Tool with Diverse Studies (QuADS) [17]. QuADS is a tool based on the 2012 Quality Assessment Tool Studies with Diverse Designs (QATSDD) [18], primarily designed to assess the quality of studies with heterogeneous designs. QuADS is an improved and revised version of QATSDD, which has demonstrated robust psychometric properties and is suitable for use in both systematic and narrative reviews. The reviewers considered that QuADS shows reliability in terms of content and face validity. QuADS assesses the quality of studies through 13 criteria related to the publication’s content. Its rating ranges from 0 (lowest quality) to 3 (highest quality). A global rating of the study’s quality is not considered since no cut-off score considers studies of high or low quality; cut-off points would be arbitrary and inappropriate. The results of the quality assessment should be discussed narratively, and the areas where the information is more or less complete and why should be considered. When ratings differ among reviewers, each will explain the reasons for their selection. For any remaining discrepancies, the scores of the three evaluators will be averaged.

## Results

### Study selection and characteristics of chosen studies

A total of 8205 articles were identified through the search in the six selected databases. After removing duplicates, 8163 remained, which went through a screening phase by title and abstract. During this screening, 8116 articles were discarded, leaving forty-seven articles for the selection phase. The full texts were reviewed based on the previously established inclusion and exclusion criteria for their final incorporation into the review; twenty-eight articles were excluded, and twenty-nine were selected. Figure 1 indicates the reasons for the exclusion of the aforementioned articles. Finally, two new articles were incorporated through the ‘snowball effect’ after searching the references of the previously selected studies. A total of twenty-one articles were included for detailed analysis (19 from the databases and two from the reference search).

Of the twenty-one included studies, the majority had experimental designs (N = 12), and the remaining nine had cross-sectional observational designs. The target population ranged from 14070 participants in a study in Canada [19] and 3097 counties in the U.S. [20] to a study with only two participants as case studies [21]. Most of the studies were conducted in Europe (N = 9) and North America (N = 7), with the rest being in Asia (N = 2), South America (N = 1), Oceania (N = 1) and Africa (N = 1).

### Analysis of the quality of the included studies

The overall scores of all the studies were high, with an average score of 29.4 and a maximum score of 35. The lowest scores were found in items 12 and 13, related to evidence that stakeholders were considered in the research design and the discussion of the studies’ limitations and strengths, revealing a lack of development in these aspects. The highest scores were seen in items related to the description of the research setting and target population and the theoretical and conceptual description of the study. Only one of the selected studies scored below 20, which could be considered of poor or low quality. View supplementary material-Table S1.

### Outcomes

The study results were grouped based on the nature-related measures’ impact on drug use, delineating into categories such as prevention, consumption/incidence, treatment, and/or mortality outcomes Table 1. Most studies focused on treatment (12), followed by incidence/consumption (7), prevention (5), and mortality (1). Additionally, the studies can be grouped based on the type of drug they focus on, with drugs, in general, being the most covered category with ten studies, followed by alcohol (6) and tobacco (6), and finally, cannabis and other drugs like opioids (4).

**Table 1:** Characteristics of the included studies in the review.

**Citation and geographic location**	**Design, sample size and population**	**Exposure ** **(Data Source)**	**Substance use domain (Drug)**	**Assessment Tools (Data source)**	**Relationship among variables**	**Main findings**
*Wiley et al (2022)* [19] Canada	Cross-sectional Study N = 14070 young adults (15–25 years)	Green Spaces (NDVI)	Incidence (Tobacco, cannabis, and alcohol)	Drug Use (CCHS)	Positive (+) and Negative (−)	Residential vegetation was not associated with overall alcohol consumption, but was associated with less frequent binge drinking (in more vegetated areas, there was an 18% reduction (OR 0.82, 95% CI [0.70–0.95]) in the odds of monthly binge drinking compared to less vegetated areas) Residential greenery was also associated with lower tobacco use (in the greenest areas, greenery was associated with a 21% decrease in the odds of being a light smoker compared to non-smokers, compared to those in the least green area (OR 0.79; 95% CI [0.68–0.93]) and increased marijuana use (in the greenest areas people had an 81% increase in the odds of being weekly users (OR 1.81, 95% CI [1.31–2.51]) and almost 50% increase in the odds of being daily users).
*Becker et al (2022)* [20] United States	Cross-Sectional Study N = 3097 counties of the U. S	Tree canopy coverage (NDVI)	Mortality (Opioids)	Mortality (CDC Data)	Negative (−)	Tree canopy coverage is positively associated with opioid-related mortality. This means that counties with higher tree canopy coverage have higher rates of opioid-related mortality (β = 0.01; p = 0.001). The association between tree canopy coverage and opioid-related mortality persists after controlling for a series of confounding factors such as urbanization level, socioeconomic status, access to healthcare, and opioid prescription rates.
*Tesler et al (2018)* [22] Israel	Experimental study N = 76 participants (Youth aged 12 to 18 at risk of dropping out of the formal education system)	Outdoor physical activity sessions (cycling, hiking, climbing…) (Questionnaires on frequency and duration of activity)	Prevention and treatment (Alcohol and Tobacco)	Alcohol, tobacco consumption, and other habits (HSBC Survey)	Positive (+)	The forest intervention program was effective in increasing physical activity (0.81 more sessions per week of 60-minute physical activities in participants compared to controls (p = 0.003)) and reducing tobacco consumption (participants decreased from an average of 2.60 (SD = 1.30) to 1.72 (SD = 1.08), while the control group increased from 3.17 (SD = 1.03) to 3.39 (SD = 1.03)) and alcohol consumption (decreased in both groups, but more in patients (−1.08 (SD = 1.30)) than in controls (−0.09 (SD = 1.79))). The forest intervention program also had a positive impact on psychosomatic symptoms (participants’ group had a change of −1.37 (SD = 0.76) versus −0.18 (SD = 0.70) in controls, respectively, with a p-value <0.001) and on adolescents’ life satisfaction (with a change of 1.42 (SD = 1.99) versus −0.29 (SD = 2.69), respectively, with a p-value = 0.013).
*Castro-Sánchez et al (2019)* [33] Spain	Cross-sectional study N = 2273 adolescents (Adolescents from the city of Granada, Spain)	Perceived motivational climate in sports and the environment where it takes place (PMCSQ-2 questionnaire and data on the nature of the environment)	Prevention and incidence (Alcohol and Tobacco)	Alcohol consumption and nicotine dependence (AUDIT test and FTDN test)	Positive (+)	Negative relationship between task climate and alcohol consumption among students who engaged in physical activities in natural environments (r = −0.121; p < 0.005). Positive relationship between ego climate and alcohol consumption among those engaged in other types of physical activity (r = 0.196; p < 0.005). Positive and direct relationship between alcohol and tobacco consumption, which was stronger among those who did not engage in physical activity in a natural environment (r = 0.497; p < 0.005).
*Wu et al (2019)* [32] Taiwan	Experimental study N = 93 participants (Daily adult smokers aged 18–30 from the city of Kaohsiung)	Exposure to natural environments (Images of natural landscapes, paintings, artworks, and urban landscapes)	Prevention and treatment (Tobacco)	Tobacco consumption, measurement of temporal discounting, and desire to smoke (Questionnaires)	Positive (+)	Participants exposed to images of natural settings smoked fewer cigarettes (mean = 1.1 cigarettes, 95% CI [(CI) = 0.7–1.6]) than those exposed to images of urban settings (mean = 2.1 cigarettes, 95% CI [1.7–2.6], p = 0.002) and participants in the control group (mean = 1.8 cigarettes, 95% CI [1.4–2.3], p = 0.029). The temporal discount rate measured the association between exposure to nature and amount of smoking (B = −0.62, 95% CI [−1.07–(−0.20)] p < 0.01).
*Dzhambov et al (2022)* [39] Austria and Italy	Cross-sectional study N = 1251 participants (Schoolchildren aged 8 to 12 years from the Tyrol Region)	Exposure to natural environments and residential gardens (D2N index and presence of gardens)	Prevention (Tobacco)	Data on tobacco consumption, concentration of cotinine/neopterin in urine, and behavioral problems (Needleman questionnaire)	Positive (+)	Children residing in homes farther away from nature exhibited greater behavioral problems at school with higher Needleman scores (β = 1.92; 95% CI: [0.43–3.41]). The effect of having a garden at home on behavioral problems acted indirectly through a lower likelihood of exposure to secondhand smoke (β = −0.03; 95% CI: [−0.05–(−0.01)]).
*Zhang et al (2023)* [34] Hong Kong	Cross-sectional study N = 1977 participants (Adult residents in Hong Kong aged 18 to 74 years)	Exposure to green spaces and parks (SVG, NDVI, and park density)	Incidence (tobacco and alcohol)	Unhealthy consumption behaviors (HKHS questionnaire)	Positive (+)	In the fully adjusted model, a higher standard deviation of SVG within a 1,000 m radius was significantly associated with lower odds of excessive alcohol consumption (OR = 0.77; 95% CI [0.62–0.95]). A higher standard deviation of SVG within an intermediate range of 400 m and 600 m was significantly associated with lower odds of excessive smoking (OR = 0.82; 95% CI: [0.68–0.99] for SVG within a 400 m intermediate range; OR = 0.80; 95% CI: [0.66–0.98] for SVG within a 600 m intermediate range). This study highlights the potential beneficial impact of residential greenness, especially in terms of street greenness, on healthier dietary habits, less binge drinking, and less excessive smoking.
*Almog et al (2022)* [35] United States	Cross-sectional study N = 340 participants (Adult residents of the U.S)	Exposure to green spaces, frequency and duration of visits, and perception of natural spaces (MTURK survey)	Incidence (Alcohol)	Alcohol consumption, data on positive and negative affect (AUDIT, PANAS-GEN, and MTURK)	Positive (+)	The product of coefficients revealed a significant indirect effect of active nature exposure on alcohol-related problems through negative affect (β = −0.048, SE = 0.022, p = 0.028), suggesting that active nature exposure may act as a protective factor against problematic alcohol consequences by reducing negative affect.
*Missen et al (2021)* [27] Australia	Quasi-experimental study N = 25 adult participants / 17 adult participants completed the questionnaire (Adults recovering from substance addiction)	Gardening therapy (Therapeutic day rehabilitation programs including an 8-month gardening component)	Treatment (Drugs in general)	Quality of life and individual semi-structured interviews (WHOQOL-BREF)	Positive (+)	Participants showed a significant improvement in their quality-of-life scores across all four dimensions after completing the program, when comparing initial and post-program scores: physical (T0 = 49.82; T1 = 61.24 (p = 0.028)), psychological (T0 = 45.29; T1 = 60.06 (p = 0.005)), and social (T0 = 46.71; T1 = 59.65 (p = 0.039)). Participants reported that the program helped them improve their physical health, psychological well-being, and social relationships by fostering friendships with others in the program.
*Masterton et al (2022)* [28] Scotland	Qualitative experimental study N = 17 participants (Adults participating in green space programs for people with PSU in Scotland and the United Kingdom)	Nature-based activity therapies (Gardening therapy, hiking, yoga…)	Treatment (General drug treatment)	Level of substance use, addiction history, and social support (Individual semi-structured interviews to explore participants’ experiences)	Positive (+)	Green space programs can be an effective intervention to support individuals with substance use problems. The results of this study can serve as a theoretical basis for such interventions, as they provide information on how to best optimize, adapt, and implement future green space programs for PSU support.
*Turunen et al (2022)* [36] Finland	Cross-sectional study N = 7321 participants (Adults from the city of Helsinki, Finland)	Exposure to residential green spaces, views from home, and frequency of visits to green spaces (Urban Atlas 2012)	Incidence (psychotropic medication)	Medication consumption and health data (Self-reported questionnaire and Helsinki Environmental Health Survey)	Positive (+)	The frequency of visits to green spaces was associated with lower odds of psychotropic medication use (OR = 0.67, 95% CI: [0.55–0.82] for 3–4 times/week; 0.78 [0.63–0.96] for ≥5 times/week). Associations between nature exposure and medication use persisted after controlling for a variety of factors, including demographic, socioeconomic, health, and behavioral factors.
*Bettmann et al (2021)* [21] United States	Experimental study N = 2 case studies (Adolescents with substance use problems)	Nature-based therapies with trained therapists (7 weeks in duration)	Treatment (Alcohol and cannabis)	Patient’s personal history, socioeconomic status, and age (Interviews and sessions with therapists)	Neutral	First case-study: Negative outcomes of nature therapy for the first case, some of the possible reasons could be lack of time in treatment and a greater sense of security in the therapeutic relationship. Second case-study: Positive outcomes of nature therapy by helping the patient experience positive emotions of pride, achievement, social skills, and a sense of belonging with peers.
*Seifert (2014)* [30] Ireland	Ethnographic experimental study N = 39 programs (Programs utilizing horticultural ecotherapy as a tool in the treatment of alcohol consumption in adults from Northern Ireland)	Nature ecotherapy interventions (NHS data)	Treatment (Alcohol)	Questionnaires/surveys to therapy coordinators and/or patients/users receiving ecotherapy.	Positive (+)	The results of this study suggest both paradigmatic and practical directions that could advance the understanding and effectiveness of ecotherapies as a treatment for alcohol addiction in the future.
*Bettmann et al (2013)* [23] United States	Experimental study N = 41 participants (Adolescents with mental and behavioral disorders, including substance abuse)	8-week interventions in nature environment (2006–2011)	Treatment (General drug treatment)	Health and drug consumption questionnaires (Y-OQ, URICA, and ARCQ)	Positive (+)	Scores at discharge were below the cut-off score of 46 or below, indicating both clinically and statistically significant improvement from treatment in total Y-OQ score (t(39) = 4.85, p < 0.001; d = 0.95, 95% CI [0.40–1.32]). Substance abuse recovery skills and readiness to change are important factors contributing to the success of therapy in nature.
*Harper et al (2019)* [24] Canada	Qualitative pilot study N = 148 participants (Adolescents with PSU aged 12–17 years)	Therapeutic rehabilitation therapies with nature experiences (Residential addiction treatment programme)	Treatment (Drugs in general)	Open-ended questionnaire with questions on benefits, challenges and self-perceived changes from therapy (Interviews and individual sessions)	Positive (+)	Adolescents who received nature therapy reported a variety of benefits, including skill development, improved self-concept, improved health, and changes in social dynamics. Adolescents identified the natural environment, outdoor activities, and interaction with therapists as key factors contributing to their success in nature therapy. Adolescents also reported some challenges, including stress, frustration, and fear.
*Richterová (2019)* [25] Czech Republic	Quasi-experimental study N = 289 participants (183 cases and 106 controls) (Participants from 6 therapeutic communities for drug addicts in the Czech Republic)	Therapeutic rehabilitation therapies with nature experiences	Treatment (Drugs in general)	Data on self-efficacy, self-esteem and stress (GSE, RES, ST)	Positive (+)	Adventure therapy was effective in the treatment of drug addiction based on GSE (Pre.Int: 29.67 vs Post.Int: 30.77), RES (Pre.Int: 18.17 vs Post.Int: 19.17) and ST (Pre.Int: 9.37 vs Post.Int: 8.77). Furthermore, the results are consistent over time, 14 days after therapy.
*Mennis et al (2021)* [37] United States	Cross-sectional study N = 126 participants (adolescents in urban settings aged 16–17 years in the Mid-Atlantic region of the U.S.)	Green space exposure (GVI and GSV)	Incidence (Drugs in general)	Data on behavior, drug use and socio-economic status (BASC-2-PRS, ASNA and DSM-5)	Positive (+)	Exposure to residential green space is associated with lower risk of substance use among urban adolescents (p < 0.005). Peer network health has a moderating effect on the relationship between residential green space exposure and substance use (p < 0.005).
*Martin et al (2020)* [38] England	Cross-sectional study N = 8059 participants (English adults from general population)	Green space exposure (LSOA)	Prevention and incidence (Tobacco)	Health, Lifestyles and Diseases Survey (HSE)	Positive (+)	After controlling for a variety of individual covariates, living in the highest green space quartile was associated with a 20% lower prevalence of current smoking compared to living in the lowest green space quartile (HR = 0.80, CI = [0.67–0.96] p < 0.017). Among ever smokers, residing in the two highest quartiles of neighbourhood green space quartiles (vs. 1st quartile) was associated with 10% and 12% higher prevalence of quitting smoking (PR = 1.10, 95% CI [1.02–1.18] p = 0.012; PR = 1.12, 95% CI [1.02–1.22] p = 0.016, respectively).
*Bittencourt et al (2023)* [26] Brazil	Experimental study N = 86 participants (Adult males aged 39.95 years from 3 therapeutic communities)	Therapeutic interventions where cooking, maintenance, gardening and gardening activities are carried out.	Treatment (Drugs in general)	Interviews with open and closed questions about the patient’s health history (Depression, Anxiety and Stress Scale-21).	Positive (+)	The participants present high levels of stress, anxiety and depression, but consider the closer contact with nature after being hosted in the Therapeutic Community as a positive factor in their rehabilitation.
*Berger & Berger (2021)* [31] Austria	Quasi-experimental study N = 20 participants and 13 controls (Adults in treatment for drug misuse)	Rehabilitation therapies in residential treatment programmes of the Anton Proksch Institute	Treatment (Alcohol)	Questionnaire on perception and preference in the choice of flora to measure and evaluate the impact of sensory perception.	Positive (+)	The sensory perception of plants can be an effective strategy to improve the well-being of residents of therapeutic communities suffering from alcohol addiction. An appropriate selection of vegetation in horticultural therapies can improve treatment outcomes in patients with alcohol addiction.
*Panagiotounis et al (2020)* [29] Greek	Quasi-experimental study N = 14 participants (Ex-drug users in treatment)	Nature therapy programme of the Therapeutic Centre for Dependents (KETHEA) in Therapeutic Communities	Treatment (Drugs in general)	Self-response survey (GSE and self-esteem scale)	Positive (+)	T-tests and effect sizes indicated that there is a statistically significant increase in participants’ self-esteem (p < 0.032, d = 0.73) and self-efficacy (p < 0.026, d = 0.93). This finding supports that adventure therapy interventions can function as an alternative or complementary therapeutic tool for use in traditional addiction recovery counselling.

**CCHS** = Canadian Community Health Survey; **NDVI** = Normalized Difference Vegetation Index; **OR** = Odd Ratio; **CI** = Confidence Interval; **CDC** = Centers for Disease Control and Prevention; **HSBC** = Health Behaviour in School-aged Children; **SD** = Standard Desviation; **PMCSQ-2** = Perceived Motivational Climate In Sport Questionnaire-2; **AUDIT** = Alcohol Use Disorders Identification Test; **FTDN** = Fagerstrom Test for Nicotine Dependence; **D2N** = Distance to Nature Index; **SVG** = Google Street View; **HKHS** = Hong Kong Health Questionnaire Unhealthy Consumption Behaviours Questionnaire; **PANAS-GEN** = Positive and Negative Affect Schedule; **MTURK** = Amazon Mechanical Turk; **WHOQOL-BREF** = World Health Organization Quality-of-Life Scale; **PSU** = Problems Substance Use; **NHS** = National Health Service in England; **Y-OQ** = Youth-Outcome Questionnaire; **URICA** = University of Rhode Island Change Assessment; **ARCQ** = Adolescent Relapse Coping Questionnaire; **GSE** = General Self-Efficacy Scale; **RES** = Rosenberg self-esteem Scale; **ST** = Stress Test; **GVI** = Green View Index; **BASC-2-PRS** = Behavior Assessment System for Children, second edition, Parent Rating Scale; **ASNA** = Adolescent Social Network Assessment; **DSM-5** = Diagnostic Statistical Manual-5; **LSOA** = Lower Layer Super Output Areas; **HSE** = Health Survey for England; **KETHEA** = Therapy Center for Dependent Individuals at Athens.

#### Treatment (N = 12)

The majority of the studies focused on exploring the link between contact with nature and its benefit in the treatment and rehabilitation of drug use in adolescents and adults. In adolescents (4 studies), nature therapy programs showed reductions in tobacco and alcohol use [21, 22], as well as improvements in emotions, social skills, self-concept, and health which supported reducing or eliminating addiction [23, 24]. In adults (8 studies), nature-based interventions were predominantly focused on general drug treatment (5 studies) [25–29], followed by alcohol addiction (2 studies) [30, 31], and one on tobacco use [32]. The nature-based therapies included activities like hiking, rafting, gardening, and viewing natural scenes. The studies showed reductions in cigarette and alcohol consumption, as well as significant improvements in quality of life, self-efficacy, self-esteem, stress levels, anxiety levels, and depression scores. This suggests the potential of nature therapies as complementary treatment options for substance abuse disorders.

#### Incidence/consumption (N = 7)

The seven cross-sectional studies evaluated the association between contact with nature, measured by vegetation indices and satellite images, and substance use and habits. In Canada, residential vegetation was associated with less frequent excessive alcohol drinking and lower tobacco consumption but higher cannabis use [19]. In Spain, alcohol and tobacco consumption were positively related, especially in those not practicing sports in natural areas [33]. In Hong Kong, higher residential greenery was associated with less likelihood of excessive alcohol and tobacco use [34]. Other studies found that nature exposure reduced negative affect, impulsive decisions, and alcohol problems [35]; it was associated with less psychotropic medication use [36], lower adolescent substance use risk, modulated by peer networks [37], and higher smoking cessation rates [38]. Overall, increased exposure to residential greenness and nature was associated with reduced alcohol and tobacco consumption and substance use problems. The exceptions were higher cannabis use in Canada and no clear associations between nature and IQ in Portugal [19, 33].

#### Prevention (N = 5)

The five studies evaluated the role of contact with nature in substance use prevention in children, adolescents, and adults through a variety of mechanisms. In adolescents, a randomized controlled trial of a nature-based sports program showed improvements in adopting healthy behaviors and protective factors against substance use [22]. This included increases in physical exercise, life satisfaction, and better mental health in the intervention group compared to the control group (p < 0.05). Another study found that among adolescents who practiced physical activity in natural environments, there was a negative relationship between task-focused climate and alcohol consumption (r = −0.121; p < 0.005) [33]. This suggests that nature contact while playing sports could contribute to lower alcohol use. Indirectly, the presence of home gardens was associated with a lower probability of secondhand smoke exposure in children (β = −0.03; 95% CI [−0.05–(−0.01)]) by being linked to less smoking among parents [39]. This indicates that nature exposure at home through gardens could prevent substance use problems in youth. In adults, an experiment exposing smokers to natural vs urban scenes found that nature visual exposure reduced cigarette consumption and desire to smoke [32]. This was mediated through the lowering of temporal discount rates between exposure and smoking amount (B = −0.62, 95% CI [−1.07–(−0.20)], p < 0.01). This suggests that nature impacts decision-making in ways that can prevent smoking.

Additionally, a study across England found that living in the greenest space quartile was associated with a 20% lower smoking prevalence compared to the lowest green quartile (PR = 0.80, CI = [0.67–0.96] p < 0.017) [38]. This further indicates that residential greenness could contribute to preventing smoking uptake in adults. In summary, the five studies indicate varied mechanisms of how contact with nature, through green spaces, home gardens, designated programs, and visual exposure, could contribute to preventing substance use and problems across age groups. The pathways were improving health behaviors, well-being, decision-making, and reducing environmental exposures.

#### Mortality (N = 1)

Only one study [20] assessed the effects of natural spaces on drug-related mortality. This study used a cross-sectional ecological design at the county level (n = 3087) in the United States. An ecological regression was used to analyze the association between tree canopy coverage and opioid-related mortality, with data from the National Land Cover Database (NLCD) for 2011 and the CDC’s WONDER database from 2008 to 2018. Stratified analyses were also conducted to examine whether the association differed by county type (metropolitan, suburban, or rural). It was found that tree canopy cover and forest cover were positively associated with opioid mortality (β = 0.01; p = 0.001). Moreover, it was found that the positive association was primarily in rural counties. These findings are unexpected because it was anticipated that exposure to green spaces would have a protective effect against opioid mortality, suggesting that the influence of green spaces on mortality from these substances may not be as direct as expected, highlighting the need for future studies or hypotheses that can explain these findings.

## Discussion

The overall results of the studies showed positive and beneficial outcomes in 85% (n = 18) of them. These results indicate the latent value of such nature-based interventions for drug addiction. In terms of specific results, the reviewed studies consistently suggest that contact with nature can have beneficial effects on reducing drug consumption and the overall well-being of individuals. Nature-based interventions such as horticulture, hiking in natural environments, and engaging in outdoor activities proved to be effective in addiction treatment and promoting recovery. Natural spaces provide open, free, and self-perceived healthy environments, which may discourage substance use as opposed to other enclosed spaces such as pubs or even the home. These findings support the idea that connection and contact with the natural world can be a valuable therapeutic resource in addiction management.

Furthermore, significant and negative associations were observed between nature contact and drug consumption, especially among young populations and adolescents. These findings suggest that regular exposure to natural environments could serve as a protective agent in preventing the onset of drug consumption, possibly by reducing associated risk factors such as stress, anxiety, and depression [40]. The consumption of drugs can have serious consequences for the physical and mental health of young people and adolescents, including neurodevelopmental disorders [41], mental disorders, cardiovascular problems [42], and the risk of overdose. Additionally, it can lead to academic underachievement [43], family and/or social problems [44], and severe economic impact [45]. Therefore, further research with longitudinal designs and longer-term programs is needed to confirm and better understand this causal relationship.

The discussion of the results obtained in this review covers several key aspects. First, it is important to highlight that 21 relevant studies were identified after a meticulous selection and evaluation process. The majority of the studies (n = 19) published since 2019 reflect a burgeoning interest in exploring the nexus between exposure to natural environments and drug use, laying a robust groundwork for identifying new trends and commonalities in this field of research.

In reviewing the categorized outcomes of the studies, a skewed distribution in research focus became apparent. There was a pronounced emphasis on treatments for addiction, overshadowing areas such as preventive strategies and the study of drug-related mortality. Probably because it’s easier to study. While this concentration on treatment is understandable given the urgency of addressing the addiction crisis, especially among youth [46], it is important to recognize the importance of addressing drug consumption from a broader perspective that includes preventive measures and harm reduction [47].

A particularly interesting aspect is the potential influence of the natural environment on drug-related mortality rates. While this topic has received less attention in the literature, the only included study suggests that certain natural environments may be associated with an increase in opioid overdose mortality rates, especially in urban areas where access to nature is limited. These findings raise important questions about the underlying mechanisms behind this association and highlight the need to consider the physical environment as a determinant of public health [48].

Exposure to natural environments can play a pivotal role in drug use prevention through a variety of social, environmental and physiological mechanisms. Emotions and stress are among the factors that are linked to the initiation, maintenance, and relapse of these habits, affecting the process of interpretation, evaluation, and response to real or perceived threatening or challenging situations and stimuli [49] and is presented as a physically and emotionally challenging process that activates adaptive responses to rebalance and achieve homeostasis within the body [50], contact with nature has been shown to improve cortisol levels improving the individual’s response to stress [51]. Social deprivation from early adulthood onwards is associated with a higher prevalence of lifetime drug abuse, contact with nature could also function as a counterbalance because it improves social cohesion. Furthermore, engaging with nature enhances emotional resilience and promotes healthy behaviors, potentially deterring substance use. Socially, nature-based activities can foster positive community interactions, providing supportive networks that diminish the appeal of drug use as a coping mechanism which can influence socio-cultural characteristics such as poverty or violence that play a major role in drug addiction [52]. Therefore, we believe that there is a need to improve the provision of green infrastructure in cities and to improve the portfolio of nature-based services by the public administration as well as quantifying, biomonitoring and/or better measuring passive/active exposure to natural areas and their influence on our health and well-being.

### Strengths and limitations

The strengths of our review include the use of many databases for the search (6) and searching for articles in two languages (English and Spanish), although only English articles were found. Additionally, it incorporates a validated and useful tool (QuADS) to measure the level of quality of heterogeneous works in this field of study. Regarding the results, it organizes exposures to nature and presents results organized by the typology of drugs and by different spheres related to substance use, facilitating interpretation and comparison. The strengths of the included studies are related to the inclusion, in a significant portion of the studies, of co-variables or confounding factors related to environmental variables, such as type of vegetation or accessibility, and sociodemographic variables, such as economic level, education, or ethnic origin, which allow for a better understanding of the results. Adequate sample sizes were used in most studies, allowing for more robust conclusions. Both qualitative and quantitative measurement tools were used. The majority used an appropriate statistical analysis method for the expected results. Lastly, they allow for the opening of future lines of research on the links between nature contact and spheres of drug use, providing valuable tools and information for administrators and community managers.

In assessing the methodological quality of studies, the variation in research designs and the demographics of the study populations must be considered. Despite the generally high scientific quality ratings, the specific limitations of each study design and the extent to which results can be generalized across different contexts should be noted. The handling of potential biases requires careful scrutiny. Our review’s limitations stem from methodological diversity, hindering data comparison and extrapolation. For this purpose, we have attempted to implement a thorough methodology for analyzing and assessing the quality of the studies. Also, the diversity of types of exposure to natural spaces also presents a limitation, as well as the lack of a clear definition of “contact with nature” in terms of variety, frequency, and time. Another limitation is the absence of appropriate search terms, such as blue spaces, that homogeneously collect important search terms. The main limitation of the reviewed studies comprising the review is that existing research still requires further confounding factors, limiting the quality of the available evidence. In addition, there may be a potential publication bias in the selected articles, as only articles with positive results are published. However, we have tried to overcome this by the most adequate studies selection.

Furthermore, data collection through self-administered surveys in many studies poses a potential bias in the results. Lack of longitudinal studies or studies conducted over longer periods of time to interpret results better and verify if intervention effects are long-lasting. Lack of control groups in some experimental intervention studies. Other limitations detected are related to the recruitment of samples and finding biases at the socioeconomic and health levels when selecting population samples with particular social and health characteristics. Nevertheless, the novel and growing nature of these types of studies using diverse methodologies and interventions provide a unique approach that allows for a wide range of results and conclusions but hinders reproducibility and comparability.

The results of this review support the idea that contact with nature can play a significant role in the prevention and treatment of drug abuse. However, further research is needed to fully understand the underlying mechanisms and to develop more effective evidence-based interventions. Additionally, it is crucial to address gaps in the literature, such as the lack of longitudinal studies, heterogeneity of methodology and the need to explore disparities in access to natural environments, to inform policies and practices that promote environmental health and the well-being of individuals and communities through the implementation of nature-based therapeutic programmes in clinical and community settings.

An integrative approach to health, incorporating nature contact into drug control and prevention strategies, requires transforming healthcare systems to promote knowledge exchange, creating new environmental medicine structures, developing new professional profiles such as environmental therapists, and increasing the involvement of citizens and environmental NGOs in mental health care and drug prevention efforts.

## Conclusion

In conclusion, this review highlights the growing interest in understanding the underexplored relationship between nature contact and drug consumption. While there is a predominant focus on addiction treatment, nature-based interventions also show promise in drug prevention and incidence, with positive results observed in most of them, such as horticultural therapies or nature adventure therapies have beneficial effects in the treatment and prevention of substance abuse based on the empirical evidence presented in the reviewed studies. Further research on the impact of nature contact on drug use is needed. Concurrently, the joint integration of health and environmental policies should encourage a comprehensive approach in national drug strategies. Furthermore, substantial alterations to municipal urban policies and an enhancement of the ‘green infrastructure’ of cities may be necessary to support these initiatives. This integrative perspective facilitates the investigation of nature-based interventions within public health frameworks, contributing to the overarching objective of planetary health.

## Supplementary information

**Additional file 1:** **Table S1.** QuADS scores breakdown by study.

## Declarations

### Availability of data and material

The data presented in this study are available on request from the corresponding author. All data used in the production of this review are included in the published studies.
