# Supplementary material for: Harnessing the healing power of nature: a review of natural interventions in substance abuse treatment and prevention
Source: Environ Health Prev Med. 2024 Nov 12;29:64. doi: 10.1265/ehpm.24-00145 (PMC11570648; doi:10.1265/ehpm.24-00145)
Supplement: Supplementary file 1 — Additional file 1: Table S1. QuADS scores breakdown by study. [file ehpm-29-064-s001.docx]

|  | Wiley et al (2022) | Becket et al (2022) | Tesler et al (2018) | Castro-Sánchez et al (2019) | Wu et al (2019) | Dzhambov et al (2022) | Zhang et al (2023) | Almog et al (2022) | Missen et al (2021) | Masterson et al (2022) | Turunen et al (2022) | Bettmann et al (2021) | Seifert (2014) | Bettmann et al (2013) | Harper et al (2019) | Richterova (2019) | Mennis et al (2021) | Martin et al (2020) | Bittencourt et al (2023) | Berger & Berger (2021) | Panagiotunis et al (2020) | **Mean score** |  |
| --- | --- | --- | --- | --- | --- | --- | --- | --- | --- | --- | --- | --- | --- | --- | --- | --- | --- | --- | --- | --- | --- | --- | --- |
| 1.Theoretical or conceptual underpinning to the research | 3 | 2 | 3 | 2 | 2 | 3 | 2 | 3 | 3 | 2 | 2 | 3 | 3 | 3 | 3 | 3 | 2 | 3 | 2 | 2 | 3 | **2,6** |  |
| 2. Statement of research aim/s | 1 | 3 | 1 | 3 | 2 | 3 | 3 | 3 | 3 | 2 | 3 | 1 | 3 | 3 | 3 | 3 | 3 | 3 | 2 | 1 | 3 | **2,5** |  |
| 3. Clear description of research setting and target population | 1 | 2 | 3 | 3 | 2 | 3 | 3 | 2 | 2 | 2 | 3 | 2 | 3 | 3 | 3 | 3 | 2 | 3 | 3 | 3 | 3 | **2,6** |  |
| 4.The study design is appropriate to address the stated research aim/s | 2 | 3 | 2 | 3 | 3 | 2 | 2 | 2 | 2 | 3 | 2 | 1 | 2 | 3 | 2 | 2 | 3 | 3 | 2 | 2 | 2 | **2,3** |  |
| 5.Appropriate sampling to address the research aim/s. | 3 | 2 | 3 | 3 | 2 | 2 | 3 | 3 | 2 | 2 | 3 | 1 | 2 | 2 | 2 | 3 | 3 | 3 | 2 | 2 | 2 | **2,4** |  |
| 6. Rationale for choice of data collection tool/s | 3 | 2 | 3 | 2 | 2 | 3 | 2 | 3 | 3 | 3 | 3 | 2 | 2 | 3 | 3 | 2 | 3 | 2 | 2 | 1 | 2 | **2,4** |  |
| 7.The format and content of data collection tools is appropriate to address the stated research aim/s | 3 | 3 | 3 | 3 | 2 | 3 | 2 | 2 | 2 | 3 | 3 | 1 | 3 | 3 | 2 | 2 | 2 | 3 | 2 | 1 | 3 | **2,4** |  |
| 8. Description of data collection process | 3 | 2 | 3 | 2 | 2 | 3 | 2 | 3 | 2 | 3 | 3 | 1 | 2 | 2 | 3 | 3 | 3 | 2 | 2 | 2 | 2 | **2,4** |  |
| 9. Recruitment data provided | 2 | 1 | 3 | 2 | 3 | 3 | 3 | 3 | 2 | 2 | 3 | 1 | 3 | 2 | 2 | 3 | 2 | 2 | 3 | 3 | 2 | **2,4** |  |
| 10.Justification for analytical method selected | 1 | 3 | 2 | 3 | 1 | 3 | 2 | 3 | 1 | 1 | 3 | 0 | 0 | 3 | 2 | 2 | 2 | 3 | 1 | 2 | 1 | **1,9** |  |
| 11.The method of analysis was appropriate to answer the research aim/s | 2 | 3 | 2 | 3 | 2 | 3 | 3 | 3 | 2 | 2 | 2 | 0 | 0 | 3 | 2 | 2 | 3 | 3 | 2 | 2 | 1 | **2,1** |  |
| 12. Evidence that the research stakeholders have been considered in research design or conduct. | 1 | 1 | 2 | 1 | 1 | 2 | 2 | 1 | 1 | 3 | 1 | 2 | 3 | 1 | 2 | 2 | 2 | 2 | 2 | 2 | 3 | **1,8** |  |
| 13. Strengths and limitations critically discussed | 2 | 2 | 2 | 0 | 0 | 3 | 2 | 2 | 2 | 2 | 3 | 1 | 0 | 2 | 2 | 2 | 3 | 3 | 1 | 0 | 2 | **1,7** |  |
| **Total score ( maximum 39)** | **27** | **29** | **32** | **30** | **24** | **36** | **31** | **33** | **27** | **30** | **34** | **16** | **26** | **33** | **31** | **32** | **33** | **35** | **26** | **23** | **29** |  |  |
| **Mean score** | **29,4** | | | | | | | | | | | | | | | | | | | | |  | |

*Table S1* . QuADS scores breakdown by study
